# Cross-sectional study on protective antibodies against influenza A virus subtypes and cross-protection against influenza A(H3N2) subclade K, Portugal, August 2025

**DOI:** 10.2807/1560-7917.ES.2026.31.6.2600070

**Published:** 2026-02-12

**Authors:** Raquel Guiomar, Camila Henriques, Susana Pereira da Silva, Licínia Gomes, Daniela Dias, Nuno Verdasca, Portuguese Laboratory Network for the Diagnosis of Influenza and Respiratory Viruses, Baltazar Nunes, Ana Paula Rodrigues, Portuguese Laboratory Network for the Diagnosis of Influenza and Respiratory Viruses, João Tiago Guimarães, Maria João Cardoso, Angelica Ramos, Paulo Pereira, Ana Paula Castro, Maria Helena Marques, Paula Mota, Ana Ferreira, Paulo Lopes, Gabriela Abreu, Adriana Pedrosa, Ana Cristina Silva, Póvoa de Varzim, Nádia Sousa Martins, Aurélio Mesquita, Marina Majar, Ana Filipa Calheiros, José António Mota Freitas, Sandra Margarida Vieira, Susana Gomes, Cacilda Magalhães, Maria J Soares, Maria José Montanha, Carlos Vaz, Helena Silva, Ezequiel Moreira, Carla Ferreira, Cristiana Lopes, Fernando Rodrigues, Teresa Reis, Sofia Almeida, Patrícia Amantegui, Tiago Costa, Patrícia Fonseca, Bruno Marques, Carina Moita, Sandra Paulo, Ricardo Rodrigues, José Afonso Rodrigues Moreira, Mariana Fradilha, Mario Cunha, Sofia Seabra, Ana Pires, Sara Correia, Diana Nunes, Cristina Toscano, Ana Paula Dias, Luís Silva, João Luís, Rita Rodrigues, Luisa Sancho, Lucia Coimbra, Elzara Aliyeva, Paula Gama, Jóni Mota, Ana Helena Lopes, Bela Nogueira, Ana Frota, Jéssica Peres, Maria João Ramos, Filomena Caldeira, Carina Rodrigues, Ana Mira, Ana Serrano, Inês Moreira, Paula Barbeiro, Elena Kronberg, Maria Paula Falcão, Alzira Louro, Armindo Gonçalves, Ana Loureiro, Teresa Garcia Rego, Maria Teresa Dias, Ana Rita Couto, Ana Marques, José Alves, Ludivina Freitas, Susana Agostinho

**Affiliations:** 1 National Reference Laboratory for Influenza and Other Respiratory Viruses, Infectious Diseases Department, National Institute of Health Dr. Ricardo Jorge, Lisbon, Portugal; 2 Department of Epidemiology, National Institute of Health Dr. Ricardo Jorge, Lisbon, Portugal; 3 The members of the Portuguese Laboratory Network for the Diagnosis of Influenza and Respiratory Viruses are listed under Collaborators and at the end of the article; 4 National School of Public Health, NOVA University, Lisbon, Portugal

**Keywords:** Influenza, seroprevalence, antibodies, A(H3N2) subclade K, cross-protection

## Abstract

The 2025/26 season was marked by co-circulation of influenza A subtypes, with the first detection of A(H3N2) subclade K in September 2025. In August 2025 in Portugal, 14.8% (95% CI: 12.2–17.8) of 886 persons tested had cross-protective antibodies against this subclade. The overall seroprevalence against circulating A(H1N1)pdm09 strains was 28.1% (95% CI: 24.4–32.0). These data highlight the presence of previous cross-reactive antibodies and the possible advantage of vaccination in the extent of detectable antibodies against influenza viruses.

The 2025/26 influenza season started earlier than the previous one, with the co-circulation of influenza A virus subtypes and the emergence of the new influenza A(H3N2) virus subclade K (former J.2.4.1 clade) observed in circulation across Europe and globally [1,2]. Here we aim to estimate the prevalence of protective antibodies against the seasonal influenza A(H1N1)pdm09 and A(H3N2) viruses, including the new subclade K, by age group and vaccination status in the previous season, in Portugal during summer 2025.

## Annual influenza serosurvey

We conducted a cross-sectional seroepidemiological survey to estimate the seroprevalence of protective antibodies against influenza A(H1N1)pdm09 and A(H3N2). In August 2025, 33 laboratories from hospitals of the mainland and the Atlantic islands of Portugal selected convenience residual sera, by age group (0–4, 5–14, 15–44, 45–64 and ≥ 65 years) and sex. Before sera anonymisation, the data on 2024/25 seasonal influenza vaccine uptake were recorded for patients aged ≥ 45 years. Sera were sent irreversibly anonymised to the National Reference Laboratory for Influenza and Other Respiratory Viruses.

We tested both influenza A strains recommended for the 2025–2026 northern hemisphere vaccine composition (A/Victoria/4897/2022 (H1N1)pdm09, clade 5a.2a.1 subclade D and A/District of Columbia/27/2023 (H3N2), clade 2a.3a.1 subclade J.2) [3] and 2025/26 circulating viruses A/Missouri/11/2025(H1N1)pdm09-like, clade 5a.2a.1 subclade D.3.1 (A/Lisboa/695/2025 EPI_ISL_20279799) and A/Norway/8765/2025(H3N2)-like, clade 2a.3a.1 subclade K (A/Lisboa/711/2025, EPI_ISL_20279659). A/Victoria/4897/2022 (H1N1)pdm09 was the vaccine strain, and it was detected in circulation with A/District of Columbia/27/2023 (H3N2) during 2024/25 season as presented in Supplementary Table S1. The viruses were grown in Mardin Darbin canine kidney cells (MDCK or MDCK-Siat1, American Type Cell Collection (ATCC), https://www.atcc.org/). All samples were analysed using haemagglutination inhibition (HAI) assay to detect antibodies against influenza viruses [4,5]. An HAI titre ≥ 40 was considered protective [4].

We estimated proportion of the population with protective antibodies against each of the studied strains, with respective 95% confidence intervals (95% CI). The overall seroprevalence estimates were age-standardised using direct method, applying age-specific seroprevalence to the European standard population [6]. Differences between groups (age and vaccination status in the previous season) in the proportion of sera with antibody titres considered protective were tested using chi square test, while differences in geometric mean titres (GMT) between groups were evaluated using the Kruskal-Wallis test. The level of significance was set at 5%. Seroprevalence and GMT ratios were calculated by age group and previous season vaccine status, using the 0–4-year-olds and the unvaccinated as reference groups. All statistical analyses were performed in R, version 4.5.1 (https://www.r-project.org).

A total of 882 sera were selected from the Portuguese population. Between 143 (16.2%) and 201 (22.8%) sera were selected from each age group (0–4, 5–14, 15–44, 45–64 and ≥ 65 years), and 426 (48.3%) were from females. Vaccination status was recorded for 62 and 94 sera from people aged 45–64 and ≥ 65 years: 46.8% (29/62) and 71.3% (67/94) samples were from vaccinated individuals, respectively. Sera selection was carried out, according to the geographical distribution of the population, as presented in Supplementary Table S2.

## Seroprevalence against influenza A(H1N1)pdm09

For all the tested samples, seroprevalence against the 2024/25 vaccine strain A(H1N1)pdm09 A/Victoria/4897/2022 (subclade D) was 19.9% (95% CI: 16.9–23.2), lower than the seroprevalence observed against the 2025/26 predominant circulating strain A(H1N1)pdm09-like A/Missouri/11/2025 (subclade D.3.1) (28.1%; 95% CI: 24.4–32.0) (Table 1). Of those with HAI ≥ 40 against A/Victoria/4897/2022, 91.4% (95% CI: 74.9–100.0) had protective antibodies against the frequently circulating A(H1N1)pdm09 from subclade D.3.1, with a GMT of 84.1 (95% CI: 56.6–103.1) (Table 2). From these, the seroprevalence and GMT were lower among persons aged ≥ 65 years: 67.9% (95% CI: 47.6–84.1) and 44.2 (95% CI: 31.0–63.0), respectively.

**Table 1:** Seroprotection against seasonal influenza A(H1N1)pdm09 (A/Victoria/4897/2022, A/Missouri/11/2025-like strain) by haemagglutination inhibition assay and geometric means titre, by age, Portugal, August 2025 (n = 882)^a^

Age group (years)	Samples (n)	A/Victoria/4897/2022^b^							A/Missouri/11/2025-like^c^						
		Positive (n)	%	95% CI	Ratio	GMT	95% CI	Ratio	Positive (n)	%	95% CI	Ratio	GMT	95% CI	Ratio
Overall^d^	882	202	19.9	16.9–23.2	NA	17.2	12.5–21.4	NA	275	28.1	24.4–32.0	NA	24.6	16.2–32.2	NA
0–4	143	29	20.3	14.0–27.8	Ref	14.1	12.3–16.2	Ref.	37	25.9	18.9–33.9	Ref.	17.9	15.0–21.4	Ref
5–14	168	82	48.8	41.0–56.6	2.4	27.3	23.5–31.6	1.9	108	64.3	56.5–71.5	2.5	44.2	37.1–52.6	2.5
15–44	201	34	16.9	12.0–22.8	0.8	12.6	11.3–14.1	0.9	52	25.9	20.0–32.5	1.0	18.8	16.2–21.8	1.1
45–64	192	29	15.1	10.4–21.0	0.7	12.1	10.9–13.4	0.9	46	24.0	18.1–30.6	0.9	16.5	14.4–19.0	0.9
≥ 65	178	28	15.7	10.7–21.9	0.8	13.7	12.3–15.3	1.0	32	18.0	12.6–24.4	0.7	15.5	13.8–17.5	0.9

CI: confidence interval; GMT: geometric means titre; NA: not applicable; Ref: reference; WHO: World Health Organization.

^a^ Haemagglutination assay (HAI) titre ≥ 40 was considered positive.

^b^ 2025/26 northern hemisphere WHO recommended vaccine strains (genetic clade 5a.2a.1 subclade D).

^c^ A/Lisboa/695/2025 EPI_ISL_20279799 (2025/26 circulating strain, genetic clade 5a.2a.1 subclade D.3.1).

^d^ The overall seroprevalence was standardised using the direct method, with the European standard population as reference.

**Table 2:** Seroprotection against seasonal influenza A/Missouri/11/2025-A(H1N1)pdm09-like and A/Norway/8765/2025-A(H3N2)-like in sera positive for A/District of Columbia/27/2023-A(H1N1)pdm09 (n = 202)^a^ or positive for A/Victoria/4897/2022-A(H3N2) (n = 560)^a^, by age, Portugal, August 2025

Age group (years)	A(H1N1)pdm09 A/Missouri/11/2025-like^b^								A(H3N2) A/Norway/8765/2025-like^c^							
	Positive (n)	Samples (n)	%	95% CI	Ratio	GMT	95% CI	Ratio	Positive (n)	Samples (n)	%	95% CI	Ratio	GMT	95% CI	Ratio
Overall^d^	189	202	91.4	74.9–100.0	NA	84.1	56.6–103.1	NA	135	560	22.3	18.1–27.1	NA	17.6	15.1-19.1	NA
0–4	28	29	96.6	82.2–99.9	Ref	99.2	70.7–139.1	Ref	22	85	25.9	17.0–36.5	Ref	16.0	13.5–19.1	Ref
5–14	81	82	98.8	93.4–100	1.0	101.4	84.7–121.3	1.0	45	135	33.3	25.5–42.0	1.3	20.7	18.0–23.9	1.3
15–44	33	34	97.1	84.7–99.9	1.0	106.4	80.0–141.7	1.1	29	124	23.4	16.3–31.8	0.9	16.7	14.7–19.0	1.0
45–64	28	29	96.6	82.2–99.9	1.0	72.7	53.2–99.4	0.7	20	100	20.0	12.7–29.2	0.8	16.5	14.2–19.2	1.0
≥ 65	19	28	67.9	47.6–84.1	0.7	44.2	31.0–63.0	0.4	19	116	16.4	10.2–24.4	0.6	14.4	12.7–16.4	0.9

CI: confidence interval; GMT: geometric means titre; NA: not applicable; Ref: reference.

^a^ Testing performed with haemagglutination assay (HAI) and titre ≥ 40 was considered positive.

^b^ A/Lisboa/695/2025 EPI_ISL_20279799 (2025/26 circulating strain, genetic clade 5a.2a.1 subclade D.3.1).

^c^ A/Lisboa/711/2025, EPI_ISL_20279659 (2025/26 circulating strain, genetic clade 2a.3a.1 subclade K).

^d^ The overall seroprevalence was standardised using the direct method, with the European standard population as reference.

Seroprevalence for both A(H1N1)pdm09 tested viruses was higher among the 5–14-year-olds (A/Victoria/4897/2022: 48.8%; 95% CI: 41.0–56.6 and A/Missouri/11/2025: 64.3%, 95% CI: 56.5–71.5) than among the 0–4-year-olds (20.3%, 95% CI: 14.0–27.8 and 25.9%, 95% CI: 18.9–33.9, respectively) (Table 1). The GMT followed the same pattern. Seroprevalence decreased then with increasing age with the lowest rates observed in people aged ≥ 65 years with 15.7% (95% CI: 10.7–21.9) against A/Victoria/4897/2022 and 18.0% (95% CI: 12.6–24.4) against A/Missouri/11/2025-like virus (Table 1).

Higher seroprevalence estimates for A(H1N1)pdm09 viruses were observed among vaccinated individuals (45–64 and ≥ 65 years), however, the estimates were not statistically significant (Table 3).

**Table 3:** Proportion of protective antibodies and geometric means titre against influenza A(H1N1)pdm09 and A(H3N2) by 2024/25 seasonal vaccination status among people aged ≥ 45 years, Portugal, August 2025 (n = 156)^a^

Influenza virus	Vaccination status	Positive (n)	Samples (n)	%	95% CI	Ratio	GMT	95% CI	Ratio
Age: 45–64 years									
A(H1N1)pdm09									
A/Victoria/4897/2022	Nvac	3	33	9.1	1.9–24.3	Ref	10.2	8.2–12.8	Ref
	Vac	7	29	24.1	10.3–43.5	2.7	13.3	9.8–18.0	1.3
A/Missouri/11/2025-like^b^	Nvac	5	33	15.2	5.1–31.9	Ref	12.9	9.7–17.1	Ref
	Vac	8	29	27.6	12.7–47.2	1.8	17.8	12.1–26.1	1.4
A(H3N2)									
A/District of Columbia/27/2023	Nvac	15	33	45.5	28.1–63.6	Ref	25.7	17.6–37.7	Ref
	Vac	13	29	44.8	26.4–64.3	1.0	33.8	20.8–55.0	1.3
A/Norway/8765/2025-like^c^	Nvac	2	33	6.1	0.7–20.2	Ref	9.6	7.2–12.7	Ref
	Vac	5	29	17.2	5.8–35.8	2.8	14.7	11.0–19.5	1.5
Age: ≥ 65 years									
A(H1N1)pdm09									
A/Victoria/4897/2022	Nvac	4	27	14.8	4.2–33.7	Ref	11.4	8.7–14.8	Ref
	Vac	11	67	16.4	8.5–27.5	1.1	13.8	11.5–16.5	1.2
A/Missouri/11/2025-like^b^	Nvac	2	27	7.4	0.9–24.3	Ref	11.7	9.0–15.1	Ref
	Vac	11	67	16.4	8.5–27.5	2.2	14.4	11.9–17.3	1.2
A(H3N2)									
A/District of Columbia/27/2023	Nvac	13	27	48.1	28.7–68.1	Ref	30.2	19.7–46.1	Ref
	Vac	44	67	65.7	53.1–76.8	1.4	39.6	31.6–49.7	1.3
A/Norway/8765/2025-like^c^	Nvac	1	27	3.7	0.1–19.0	Ref	10.5	8.6–12.9	Ref
	Vac	10	67	14.9	7.4–25.7	4.0	13.5	11.3–16.1	1.3

CI: confidence interval; GMT: geometric means titre; Nvac: non vaccinated; Ref: reference; Vac: vaccinated.

^a^ Testing performed with haemagglutination assay (HAI) and titre ≥ 40 was considered positive.

^b^ A/Lisboa/695/2025 EPI_ISL_20279799 (2025/26 circulating strain, genetic clade 5a.2a.1 subclade D.3.1).

^c^ A/Lisboa/711/2025, EPI_ISL_20279659 (2025/26 circulating strain, genetic clade 2a.3a.1 subclade K).

## Seroprevalence against influenza A(H3N2) virus and cross-protection to the new influenza A(H3N2) subclade K

The highest prevalence of protective antibody titres was detected for A/District of Columbia/27/2023 (subclade J.2) (61.8%; 95% CI: 56.2–67.7), a frequently detected strain in the late months of the previous influenza season. Seroprevalence of protective antibodies against the new A(H3N2) subclade K was 14.8% (95% CI: 12.2–17.8), significantly lower than the seroprevalence against the J.2 subclade strain (Table 4). The GMT reflected the same pattern. Of those with HAI ≥ 40 against A/District of Columbia/27/2023, 22.3% (135/560) had a protective antibody titre against the A(H3N2) subclade K. Among these individuals, seroprevalence was highest in children and adolescents aged 5–14 years (33.3%; 95% CI: 25.5–42.0) and lowest in people aged ≥ 65 years (16.4%; 95% CI: 10.2–24.4) (Table 2).

**Table 4:** Seroprotection against seasonal influenza A(H3N2) (A/District of Columbia/27/2023 and A/Norway/8765/2025-like strain) by haemagglutination inhibition assay and geometric means titre, by age, Portugal, August 2025 (n = 882)^a^

Age group (years)	Samples (n)	A/District of Columbia/27/2023^b^							A/Norway/8765/2025-like^c^						
		Positive	%	95% CI	Ratio	GMT	95% CI	Ratio	Positive	%	95% CI	Ratio	GMT	95% CI	Ratio
Overall^d^	882	560	61.8	56.2–67.7	NA	49.8	33.2–62.9	NA	140	14.8	12.2–17.8	NA	14.3	12.2–15.6	NA
0–4	143	85	59.4	50.9–67.6	Ref	40.0	32.3–49.5	Ref	22	15.4	9.9–22.4	Ref	12.3	10.9–13.8	Ref
5–14	168	135	80.4	73.5–86.1	1.4	83.3	69.7–99.5	2.1	45	26.8	20.3–34.2	1.7	17.5	15.4–19.9	1.4
15–44	201	124	61.7	54.6–68.4	1.0	36.6	31.7–42.2	0.9	31	15.4	10.7–21.2	1.0	13.0	11.8–14.4	1.1
45–64	192	100	52.1	44.8–59.3	0.9	30.7	26.3–35.9	0.8	22	11.5	7.3–16.8	0.7	12.1	10.9–13.4	1.0
≥ 65	178	116	65.2	57.7–72.1	1.1	38.5	33.4–44.4	1.0	20	11.2	7.0–16.8	0.7	12.4	11.3–13.6	1.0

CI: confidence interval; GMT: geometric means titre; NA: not applicable; WHO: World Health Organization.

^a^ Haemagglutination assay (HAI) titre ≥ 40 was considered positive.

^b^ 2025/26 northern hemisphere WHO recommended vaccine strains (clade 2a.3a.1 subclade J.2).

^c^ A/Lisboa/711/2025, EPI_ISL_20279659 (2025/26 circulating strain, genetic clade 2a.3a.1 subclade K).

^d^ The overall seroprevalence was standardised using the direct method, with the European standard population as reference.

The seroprevalence of protective antibodies against A/District of Columbia/27/2023) was > 50% in all age groups (Table 4). The highest seroprevalence was found in children and adolescents aged 5–14 years (80.4%; 95% CI: 73.5–86.1), which was 1.4 times higher than in the youngest children (0–4 years: 59.4%; 95% CI: 50.9–67.6). Prevalence in people aged ≥ 15 years varied between 52.1 and 65.2%.

For the new influenza A(H3N2) subclade K, the highest seroprevalence was also observed in children and adolescents aged 5–14 years, at 26.8% (95% CI: 20.3–34.2). Seroprevalence of protective antibodies against the A(H3N2) subclade K was lower in people aged 45–64 and ≥ 65 years, at 11.5% (95% CI: 7.3–16.8) and 11.2% (95% CI: 7.0–16.8), respectively. The GMT followed the same pattern (Table 4).

Among vaccinated individuals in the previous season (2024/25), seroprevalence against the A(H3N2) subclade K was higher than in the non-vaccinated individuals in the 45–64 and ≥ 65 years age groups. Differences should be interpreted with caution due to the uncertainty of the estimates.

## Discussion

This annual serosurvey provides population-level estimates of antibody seroprevalence against influenza A(H1N1)pdm09 and A(H3N2) in August 2025, in Portugal.

The study demonstrated cross-reactive antibodies against the newly emerged A(H3N2) subclade K viruses, as well as protective antibodies against circulating influenza A viruses.

In the tested population, the highest proportions of protective antibodies were measured against influenza A(H3N2) (A/District of Columbia/27/2023, J.2 subclade) (61.8%) followed by the A(H1N1)pdm09 circulating strain (A/Missouri/11/2025, D.3.1 subclade) (28.1%). These findings align with the late spring circulation of influenza virus and the predominant viruses in the previous season. In Portugal, 2024/25, the A(H3N2) was predominantly detected in 40.4% of influenza cases, co-circulating with the A(H1N1)pdm09 in 16.5% of the cases [7]. Most A(H3N2) viruses in the previous season belonged to the J.2 subclade, which is ancestral to the K subclade [7]. The A(H1N1)pdm09 viruses were mainly from the C.1.9 subclade, which differs from the circulating viruses in the present season, although they are highly antigenically similar despite their different genetic origins [7,8].

In Portugal, the 2025/26 season started with predominant circulation of A(H1N1)pdm09 accounting for 63% of influenza cases from week 40/2025 to week 1/2026 [9]. The A(H3N2) subclade K was detected in September 2025 (week 36/2025) with increased detections in December 2025 [9]. Our study found that 14.8% of the overall population had pre-existing cross-reactive antibodies against A(H3N2) subclade K. Of those with a protective antibody titre against the A(H3N2) vaccine virus, 22.3% (135/560) had a protective titre against the subclade K, highlighting the pre-season antibodies against a new influenza subclade [3].

Differences in the prevalence of protective antibodies against influenza A viruses were observed across age groups and vaccination status in 2024/25. The highest seroprevalence and GMT against A(H1N1)pdm09 and A(H3N2) were observed in children and adolescents aged 5–14 years. This is probably linked to the highest proportion of confirmed influenza A cases in this age group in the previous season [7]. In Portugal, influenza vaccination in children and adolescents is recommended only for those aged > 6 months with chronic diseases [10], so the observed seroprevalence likely reflects antibodies due to previous infection. This highlights the role of school-aged children and adolescents in influenza transmission within the population, as observed in previous seasons [5]. The lowest seroprevalence and GMT were observed in adults aged ≥ 45 years. Seroprevalence against the A(H3N2) subclade K viruses was higher among those aged 5–14 years and lower in adults aged ≥ 45 years.

In Portugal, influenza vaccination is recommended for people aged ≥ 60 years [10]. In the 2024/25 season, egg-based trivalent vaccine (TIV) (< 85 years) and high-dose TIV (≥ 85 years) were available with a coverage of 64.0% for persons aged ≥ 60 years and 85.1% for those aged ≥ 85 years [11]. High vaccination coverage in these age groups, coupled with the exposure to circulating influenza A viruses during the late 2024/25 season (February–May 2025), may have contributed to maintaining detectable protective antibodies against influenza A for 10 months after the vaccination campaign. Although higher point estimates against the new A(H3N2) subclade K were observed among vaccinated individuals, the uncertainty around the estimates did not allow firm conclusions regarding differences between vaccinated and non-vaccinated groups. Further studies are needed to support the possibility that vaccination can elicit cross‐reactive antibody responses against future emerging strains [12]. Early estimates on influenza vaccine effectiveness (VE) against influenza-related medical encounters and hospitalisations in studies from European countries showed a high to moderate VE [13-15]. These estimates are consistent with ours and may suggest that the current influenza vaccines are providing protection against the circulating viruses.

The results of our study must be interpreted with caution and in the light of the following limitations: the sample selection was not randomised; however, samples were collected from several laboratories from hospitals covering the geographical diversity of Portugal mainland and Atlantic islands. The previous influenza infection was not measured, and vaccination status was recorded only for individuals aged ≥ 45 years. Finally, the HAI assay assesses antibodies that inhibit haemagglutinin receptor but does not detect other antigen specific antibodies, nor evaluate the cellular immune response.

## Conclusion

We estimated the seroprevalence of protective antibodies against influenza A viruses, during the summer, before the 2025/26 season. The evidence of cross-reactive antibodies against the new influenza A(H3N2) strain from subclade K, which may influence the transmission dynamics of these viruses currently circulating in Portugal and the European region with implications on population susceptibility, should be considered in public health risk assessment and preparedness. Vaccination appeared to confer broader antibody response against circulating and newly influenza strains which is crucial for guiding vaccination plans and public health interventions.

## Data Availability

Sequences from the viruses used in the haemagglutination inhibition assays can be retrieved from GISAID using the EPI_ID: EPI_ISL_20279799 (A/Lisboa/695/2025) and EPI_ISL_20279659 (A/Lisboa/711/2025).

## Members of the Portuguese Laboratory Network for the Diagnosis of Influenza and Respiratory Viruses with affiliations

São João Local Health Unit: João Tiago Guimarães, Maria João Cardoso, Angelica Ramos; Santo António Local Health Unit: Paulo Pereira, Ana Paula Castro, Maria Helena Marques; Alto Ave Local Health Unit: Paula Mota, Ana Ferreira; Gaia e Espinho Local Heath Unit: Paulo Lopes, Gabriela Abreu; Entre Douro e Vouga Local Health Unit: Adriana Pedrosa, Ana Cristina Silva; Póvoa de Varzim; Vila do Conde Local Health Unit: Nádia Sousa Martins; Braga Local Health Unit: Aurélio Mesquita, Marina Majar; Alto Minho Local Health Unit: Ana Filipa Calheiros, José António Mota Freitas, Sandra Margarida Vieira; Matosinhos Local Health Unit: Susana Gomes, Cacilda Magalhães, Maria J Soares; Nordeste Local Health Unit: Maria José Montanha, Carlos Vaz; Médio Ave Local Health Unit: Helena Silva, Ezequiel Moreira, Carla Ferreira; Coimbra Local Health Unit: Cristiana Lopes, Fernando Rodrigues, Teresa Reis; Cova da Beira Local Health Unit: Sofia Almeida, Patrícia Amantegui; Guarda Local Health Unit: Tiago Costa, Patrícia Fonseca, Bruno Marques, Carina Moita; Castelo Branco Local Health Unit:: Sandra Paulo, Ricardo Rodrigues; Baixo Mondego Local Health Unit: José Afonso Rodrigues Moreira, Mariana Fradilha; Portuguese Institute of Oncology Francisco Gentil, Lisbon: Mario Cunha; Arrábida Local Health Unit: Sofia Seabra, Ana Pires, Sara Correia, Diana Nunes; Lisboa Ocidental Local Health Unit: Cristina Toscano, Ana Paula Dias; Estuário do Tejo Local Health Unit: Luís Silva, João Luís, Rita Rodrigues; Amadora and Sintra Local Health Unit: Luisa Sancho, Lucia Coimbra, Elzara Aliyeva; Médio Tejo Local Health Unit: Paula Gama, Jóni Mota, Ana Helena Lopes; Arco Ribeirinho Local Health Unit: Bela Nogueira, Ana Frota, Jéssica Peres, Maria João Ramos; Alentejo Central Local Health Unit: Filomena Caldeira, Carina Rodrigues, Ana Mira, Ana Serrano, Inês Moreira; Litoral Alentejano Local Health Unit: Paula Barbeiro, Elena Kronberg; Alto Alentejano Local Health Unit: Maria Paula Falcão, Alzira Louro; Laclibe - Beja Clinical Analysis Laboratory: Armindo Gonçalves; Algarve Local Health Unit: Ana Loureiro; Ponta Delgada Divino Espírito Santo Hospital - São Miguel Island, Azores: Teresa Garcia Rego, Maria Teresa Dias; Santo Espírito Hospital, Terceira Island, Azores: Ana Rita Couto; Horta Hospital, Azores: Ana Marques; Hospital Dr. Nélio Mendonça, Madeira Island: José Alves, Ludivina Freitas; Laboratório Synlab Madeira: Susana Agostinho.

## Supplementary Data

634000 Supplementary Material
