# Supplementary material for: Cross-sectional study on protective antibodies against influenza A virus subtypes and cross-protection against influenza A(H3N2) subclade K, Portugal, August 2025
Source: Euro Surveill. 2026 Feb 12;31(6):2600070. doi: 10.2807/1560-7917.ES.2026.31.6.2600070 (PMC12905529; doi:10.2807/1560-7917.ES.2026.31.6.2600070)
Supplement: Supplementary Material [file 26-00070_GUIOMAR_Supplement.pdf]

Supplementary Material

This supplementary material is hosted by *Eurosurveillance* as supporting information alongside the article **Cross-sectional study on protective antibodies against influenza A virus subtypes and cross-protection against influenza A(H3N2) subclade K, Portugal, August 2025**, on behalf of the authors, who remain responsible for the accuracy and appropriateness of the content. The same standards for ethics, copyright, attributions and permissions as for the article apply. Supplements are not edited by *Eurosurveillance* and the journal is not responsible for the maintenance of any links or email addresses provided therein.

Supplementary Table S1. Influenza virus strains from A(H1N1)pdm09 and A(H3N2) subtypes tested in the hemagglutination inhibition assay.

Influenza virus			2024/25		2025/26	
Subtype	Strain	Genetic clade (subclade)	Vaccine composition	In circulation	Vaccine composition	In circulation
A(H1N1)pdm09	A/Victoria/4897/2022	5a.2a.1 (D)	●	●	●	
A(H3N2)	A/District of Columbia/27/2023	2a.3a.1 (J.2)		●	●	
A(H1N1)pdm09	A/Missouri/11/2025	5a.2a.1 (D.3.1)				●
A(H3N2)	A/Norway/8765/2025	2a.3a.1 (K)				●

Supplementary Table S2. Demographic characteristics of the sera selected and tested by hemagglutination inhibition assay to assess the seroprevalence of protective antibodies against influenza A viruses, summer 2025, Portugal.

	n/N (%)
**Age group**	
**0-4**	143/882 (16.2%)
**5-14**	168/882 (19.0%)
**15-44**	201/882 (22.8%)
**45-64**	192/882 (21.8%)
**65+**	178/882 (20.2%)
**Sex**	
**Female**	426/877 (48.6%)
**Male**	451/877 (51.4%)
**Region**	
**Norte**	148/882 (16.8%)
**Centro**	138/882 (15.6%)
**LVT**	150/882 (17.0%)
**Alentejo**	133/882 (15.1%)
**Algarve**	50/882 (5.7%)
**Açores**	128/882 (14.5%)
**Madeira**	135/882 (15.3%)
**Vaccination status 2024/25**	
**45-64 vacinated**	29/62 (46.8%)
**65+ vaccinated**	67/94 (71.3%)
